# Intra- and interspecific ecophysiological responses to waterlogging stress in two contrasting waterlogging-tolerant arbor species

**DOI:** 10.3389/fpls.2023.1257730

**Published:** 2023-11-13

**Authors:** Mengjie Tian, Dadong Li, El-Hadji Malick Cisse, Lingfeng Miao, Jingjing Zhou, Weizong Yang, Boshen Chen, Lijun Li, Huimin Tian, Bingbing Ye, Fan Yang

**Affiliations:** ^1^ Key Laboratory of Agro-Forestry Environmental Processes and Ecological Regulation of Hainan Province, Center for Eco-Environment Restoration Engineering of Hainan Province, School of Ecological and Environmental Sciences, Hainan University, Haikou, China; ^2^ School of Life Sciences, Hainan University, Haikou, China; ^3^ School of Plant Protection, Hainan University, Haikou, China

**Keywords:** competition, facilitation, forest species, inter-specific, intra-specific, neighboring relationship, waterlogging

## Abstract

At present, establishing planted forests, typically composed of not more than two tree species, to avoid forest losses has received increasing attention. In addition, investigating the impact of environmental stress such as waterlogging on different planting patterns is essential for improving wetland ecosystem resilience. Knowledge about the impact of waterlogging on planted forests is crucial for developing strategies to mitigate its adverse effects. Here, we conducted experimentally a simulated pure and mixed planting system composed of two contrasting WL-tolerant species (*Cleistocalyx operculatus* and *Syzygium cumini*) to determine their ecophysiological responses based on the type of interaction. Results showed that the aboveground growth performance of *S. cumini* was better than that of *C. operculatus* under well-watered conditions regardless of the planting model, which is contrary to the belowground accumulation that was significantly improved in *C. operculatus*. Intra- and interspecific interactions in different planting models facilitated the growth performance of *C. operculatus* while provoking a significant competition in *S. cumini* under waterlogging. Such phenomenon was explained through the remarkable ability of *C. operculatus* to naturally increase its root network under stress on non-stress conditions compared with *S. cumini*. In this study, two main factors are proposed to play key roles in the remarkable performance of *C. operculatus* compared with *S. cumini* following the planting model under waterlogging. The high level of nitrogen and phosphor absorption through *C. operculatus* primary roots and the significant starch biosynthesis constituted the key element that characterized the facilitation or competition within the intra- or interspecific interactions shown in *C. operculatus* compared with *S. cumini*. Furthermore, the intraspecific competition is more pronounced in *S. cumini* than in *C. operculatus* when grown in a pure planting pattern, particularly when subjected to waterlogging. However, when the two species are planted together, this competition is alleviated, resulting in enhanced waterlogging tolerance.

## Introduction

Plant–plant interactions play a crucial role in regulating plant growth, species coexistence, and community composition (Wang and Li, 2016). Numerous studies have focused on competition as the most crucial interaction among neighboring plants (Zhang et al., 2013; Li et al., 2023b). In general, competition intensifies when significant similarities are found in resource needs (Levine and HilleRisLambers, 2009) or when plants exhibit kin recognition (Mahall and Callaway, 1992; Murphy and Dudley, 2009). Plant neighboring could trigger an increased investment into competitive organs (Poorter et al., 2012). For example, in the presence of *Suaeda salsa*, *Phragmites australis* allocated more biomass into the roots for soil water absorption (Guo et al., 2022). However, positive plant–plant interactions (facilitation) have been widely explored in the past two decades (Bertness and Callaway, 1994). In addition, positive interactions among plants under harsh conditions can create a beneficial environment that supports the growth of certain species, leading to the potential expansion of their geographic range (Bulleri et al., 2016; Filazzola et al., 2018). Plant–plant interactions can be altered possibly because of abiotic factors (e.g., water, nutrients, light, or space) and neighbor or target plant characteristics (Pennings et al., 2003; Li et al., 2015). Hence, obtaining a comprehensive understanding of the changes occurring in plant interactions is crucial when exposed to external abiotic factors such as waterlogging and in the presence of neighboring plants.

Waterlogging impedes gas exchange between the soil and atmosphere, leading to energy deficiency in plant roots and accumulation of toxic metabolites and reactive oxygen species (ROS) (Subbaiah and Sachs, 2003). In addition, waterlogging interferes with plant growth (Parad et al., 2013), reducing root activity (Li et al., 2022) and affecting the photosynthesis (Yordanova et al., 2005; Yordanova and Popova, 2007), uptake, and transport of mineral elements (Patrick et al., 1985; Pezeshki, 2001); nutrient distribution patterns (Smethurst et al., 2005; Pierce et al., 2010); and other physiological processes, thereby leading to plant death. Nevertheless, plants do not passively accept the damages caused by waterlogging stress. Plants exhibit various morphological and physiological responses mainly through “quiescence” and “escape” strategies (Garssen et al., 2015). This phenomenon has been observed in *Crataeva tapia* L. (Capparaceae) seedlings, hypertrophied lenticels, and adventitious roots (ARs) during waterlogging (Parolin, 2001a; Parolin, 2001b). Moreover, these species undergo leaf shedding and produce new leaves as a response to waterlogging (Parolin, 2002), indicating their resilience to prolonged waterlogging. In tropical areas, summer waterlogging is a factor influencing plant–plant interactions, which often represent an important bottleneck for species survival. The frequency and intensity of future extreme rainfall events will continue to increase as global climate change intensifies (Kreuzwieser and Rennenberg, 2014). Consequently, such an increase may have further impacts on plant–plant interactions. A previous study has shown that the interspecific interactions between *Phragmites australis* and *Spartina alterniflora* varied under environmental conditions, and the intensity of competition was affected by the level of the tidal zones (Yuan et al., 2013). Yang et al. (2022) reported that waterlogging induced the competitive relationship under well-watered conditions into a mutualistic relationship between *Cleistocalyx operculatus* and *Syzygium jambos*; both species showed improved tolerance to waterlogging stress. By contrast, Yue et al. (2019) discovered that waterlogging on dry lands can significantly improve the competitiveness of invasive *Bidens pilosa* L. over native *Bidens biternate* (Lour.) Merr. These disparate findings imply an incomplete comprehension of the mechanisms governing plant–plant interactions in the presence of waterlogging.

*Syzygium cumini* and *C. operculatus* are medicinal tropical terrestrial tree species belonging to the family Myrtaceae, which are primarily distributed in South China and other tropical areas (Li et al., 2022). Our previous studies confirmed that both species are waterlogged tolerant, and *C*. *operculatus* showed more tolerance to waterlogging than *S. cumini* (Li et al., 2022; Li et al., 2023a; Li et al., 2023b; Li et al., 2023c). However, the effects of waterlogging stress on the neighboring relationships between *C. operculatus* and *S. cumini* remain unexplored. *Cleistocalyx operculatus* and *S. cumini* are forest species that might be suitable in different wetland and riparian forest areas. They can be used in different planted forest systems (mixed or pure planted forest). Planted forests have emerged as a viable solution to combat deforestation and promote sustainable land use. By carefully selecting and planting specific tree species, these forests can restore and preserve crucial ecosystem functions. Nevertheless, the successful establishment and long-term survival of planted forests rely heavily on the intricate relationship among different selected tree species. Despite the growing emphasis on establishing planted forests, typically composed of not more than two tree species, to avoid forest losses, the characterization and understanding of the intra- or interspecific interactions among plant species under abiotic stresses such as waterlogging are still lacking. The key concern in planted forests in wetland ecosystems is the limited knowledge regarding the mechanism by which tree species interact with one another to establish their adaptation and resilience against submergence conditions. Here, we provided a rare study that deciphers the ecophysiological responses of tree species against waterlogging and the type of planting pattern based on a comprehensive understanding of how different tree species interact with one another with regard to resource competition and facilitation. By unraveling the intricacies of these interactions, researchers and forest managers can make informed decisions regarding tree species selection, planting patterns, and forest management practices, thereby enhancing the resilience and productivity of planted forests. Furthermore, characterizing the interactions within planted forests can shed light on potential synergies and trade-offs among different tree species. Thus, several hypotheses have been raised: Does the combination of *C. operculatus* and *S. cumini* species exhibit cooperative relationships, where they mutually benefit from each other’s presence, leading to enhanced growth and survival? Is it possible that the combination of *C. operculatus* and *S. cumini* may result in competition for resources or negative interactions, which could hinder their productivity?

## Materials and methods

### Plant materials and experimental designs

Two-year-old saplings of *C. operculatus* and *S. cumini* were collected from a local commercial tree nursery in February 2022. We cut each sapling at 5 cm above the soil surface to ensure uniform growth after re-tillering. The roots were washed carefully with tap water. In this study, well-watered and waterlogging treatments were used, and the three planting patterns were monocultures, pure planting, and mixed planting. For each species, the well-watered and waterlogging treatments were marked as CK-S and WL-S, respectively, in the single planting pattern; CK-P and WL-P, respectively, in the pure planting pattern; and CK-M and WL-M, respectively, in the mixed planting pattern. Two saplings were planted 8 cm apart in each pot (10 L, upper bore 258 mm, lower bore 230 mm × 270 mm high), and each pot was filled with 8 kg of soil (red soil:sand = 2:1, v/v). The single plant was planted in a 5-L pot, which was filled with 4 kg of soil (upper bore 225 mm, lower bore 205 mm × 150 mm high). All the treatments were placed in the experimental greenhouse at Hainan University (20°03′33.2″N, 110°20′16.9″E). The area has a typical tropical monsoon climate, and healthy saplings with almost uniform growth were selected for the waterlogging experiment after 3 months.

Waterlogging treatment started on 17 May 2022, and the water level of the mixture and monoculture plants was maintained at 5 cm above the soil surface. A total of 15 saplings (five biological replications, each with at least three saplings) were used for each treatment in each species, and the waterlogging treatment ended up on 27 August 2022. The treatments lasted for 130 days.

### Analysis of biomass accumulation

At the end of the waterlogging experiment, we collected and recorded the stem height increment (SHI), adventitious root fresh weight (ARFW), primary root fresh weight (PRFW), stem fresh weight (SFW), leaf fresh weight (LFW), total biomass (TB), and the ratio of aboveground to belowground fresh weight (A/B). The total leaf area (TLA) in each plant was determined using the LI-3000 C Area Meter (LI-COR Inc., USA).

### Determination of gas exchange and chlorophyll content

On 15–16 August 2022, the net photosynthetic rate (*A*), stomatal conductance (*g*_s_), and transpiration rate (*E*) were measured on the youngest, fully expanded leaves of each sapling with an open gas exchange system (LI-6400XT, LI-COR Inc.) between 08:30 a.m. to 11:30 a.m. In ensuring the scientific accuracy of the measurement, the leaf temperature was set at 28°C, the optical quantum flux density was set at 1,500 μmol m^−2^ s^−1^, and the relative humidity was controlled at approximately 65%–70%. The chlorophyll contents were extracted using 80% (v/v) chilled acetone. The absorbance of chlorophyll a (*Chla*), chlorophyll b (*Chlb*), and carotenoids (*Caro*) was recorded at 663, 646, and 470 nm, respectively. The total chlorophyll (*TChl*) was calculated as the sum of *Chla* and *Chlb* (Li et al., 2023a).

### Determination of the emergence time, activity, lignin content of AR, and porosity of primary roots

During the experiment, the emergence time of ARs as the point at which root primordia on the stems of waterlogged plants measured ≥5 mm was recorded. For each plant, we meticulously recorded the emergence time of AR. The root porosity (% of the volume of gas per unit of tissue volume) was determined at the end of the experiment in accordance with the method of Munir et al. (2019). The weight biomass recorded above and the formula proposed by Jensen et al. (1969) were used to calculate root porosity:


$$Porosity\;\left(\%\right)\;=\;100\times {\;}^{*}\;\left(M_{\text{h}}-M_{\text{a}+\text{b}}\right)\;/\;(M_{\text{b}}+M_{\text{a}}-M_{\text{a}+\text{b}}),$$

where *M*_b_ is the mass of the water-filled specific gravity bottle, *M*_a_ is the ARFW, *M*_a+b_ is the mass of ARs placed into the water-filled specific gravity bottle, and *M*_h_ is the mass of the specific gravity bottle filled with the homogenate of ARs.

The lignin contents of ARs were measured using the acetyl bromide method (Li et al., 2022). The absorbance was determined at 280 nm using a spectrophotometer, and the unit of lignin content was expressed as *A*_280_·g^−1^·FW. The root activity of ARs and primary roots was measured using the method described by Li et al. (2022). The absorbance was recorded at 485 nm and compared with that of the calibration curve, and the root activity was expressed as mg·g^−1^·h^−1^·FW.

### Calculation of competitive relationships

The relative interaction intensity (RII), relative competition intensity (RCI), and aggressivity between *C. operculatus* and *S. cumini* in different planting patterns were calculated separately to determine the competitive relationship. The calculations were based on the following formula:


$$\text{RII }=\frac{Pw-Ps}{Pw+Ps}$$

where *P_W_* and *P_S_* are the performance of plants with and without neighbors, respectively. The formulas used were in accordance with the method of Zhang et al. (2020).


$$\text{RCI }=\frac{Y_{\text{aa}-}Y_{\text{ab}}}{Y_{\text{aa}}}$$

$$A=\;-\frac{Y_{\text{ab}}}{Y_{\text{aa}\times }Z_{\text{ab}}}-\frac{Y_{\text{ba}}}{Y_{\text{bb}\times }Z_{\text{ba}}}$$

where a and b represent *S*. *cumini* and *C. operculatus*, respectively; *Y*_ab_ and *Y*_ba_ represent the mixed planting biomass of *S*. *cumini* or *C. operculatus*; and *Y*_aa_ and *Y*_bb_ represent the same species planting biomass. *Z*_ab_ and *Z*_ba_ are the proportion of *S*. *cumini* and *C. operculatus* in the mixed planting system, and *A* indicates aggressivity. In addition, the RCI was analyzed in accordance with the method of Grace (1995), and aggressivity was calculated in accordance with the method of McGilchrist and Trenbath (1971).

### Determination of soluble protein, proline, peroxidase, ascorbate peroxidase, and superoxide dismutase

All operations were performed at 4°C. Cells (0.2 g) of plant leaves were homogenized in a mortar with 5 mL of 50 mM phosphate buffer at pH 7. The filtrate was centrifuged at 15,000×*g* for 15 min at 4°C. Soluble protein, free proline, peroxidase (POD), and superoxide dismutase (SOD) were measured as described by our previous reports (Yang et al., 2022). Ascorbate peroxidase was measured as described by Lin and Pu (2010).

### Determination of superoxide radical, malondialdehyde, soluble sugar, starch, and midday leaf water potential

Superoxide radical (O_2_^·−^), malondialdehyde (MDA), soluble sugar, and starch were measured as described by our previous reports (Li et al., 2022). Midday leaf water potential (*Ψ* md) was measured in the leaf used for the determination of gas exchange and chlorophyll fluorescence using a potentiometer (WP4C; Decagon Devices, Inc., Pullman, WA, USA) in accordance with a previously described protocol (Liao et al., 2019).

### Determination of soil and root mineral element content

At the end of the experiment, the soil and roots from CK and waterlogging treatments were collected and then oven-dried at 80°C for 72 h to a constant weight. The samples were ground in a mortar and passed through a 100-mesh sieve. Approximately 0.5 g of powdered soil and 0.1 g of root sample were digested with 5 mL H_2_SO_4_ for the determination of total nitrogen and total phosphorus contents by using the semi-micro-Kjeldahl method and then determined by using a fully automated flow analyzer (PROXIMA 1022/1/1, ALLIANCE Instruments, France).

### Statistical analysis

SPSS 25.0 (SPSS, Chicago, IL, USA) was used to perform statistical analyses. Data were checked for normality and homogeneity of variances before the analysis and Ln-transformed if these assumptions were not satisfied. One-way ANOVAs were used to determine differences between the two treatments, and Duncan’s multiple range test was employed to detect possible differences among means. An independent-sample *t*-test was used to compare the differences between the two species. Differences and correlations were considered to be significant at *P<*0.05.

Structural equation modeling (SEM) analysis was performed using IBM SPSS AMOS Ver. 26. The chi-square test (*χ* ^2^) was used to test the overall fit of the SEM. If the model fits the *χ* ^2^/*df* index between 0.00 and 2.00 and the *P*-value is greater than 0.05, then the model was considered acceptable. Based on the Bonferroni correction, the correlation of the probability level is at *P*< 0.01 = 0.05/5, which was considered to be significant by SEM analysis. The root mean square error of approximation, the comparative fit index, Tucker–Lewis’s index, and the optimal range of values are as described by Fan et al. (2016).

## Results

### Comparative analysis of the morphological traits and biomass accumulation between *Syzygium cumini* and *Cleistocalyx operculatus* among the treatments

Significant interspecific differences between the levels of TB and A/B were found in all treatments (**Table 1**). Compared with the CK-S treatment, the CK-P treatment significantly decreased the SHI, PRFW, SFW, TLA, LFW, and TB in *S*. *cumini*, as well as the TLA, LFW, and A/B in *C. operculatus*, but significantly increased the A/B in *S*. *cumini* and the PRFW in *C. operculatus*. The CK-M treatment significantly decreased the SHI, PRFW, SFW, TLA, LFW, and TB in *S*. *cumini*, but not in *C. operculatus*. In addition, compared with the CK-P treatment, the CK-M treatment significantly increased the PRFW, TLA, LFW, and TB in both species and the SFW in *C. operculatus*. The A/B significantly decreased in both species. Furthermore, compared with the WL-S treatment, the WL-P treatment significantly decreased the SHI in both species, as well as the ARFW, PRFW, SFW, TLA, LFW, and TB in *S*. *cumini*, but increased the A/B in *S*. *cumini*, as well as the PRFW, LFW, and TB in *C. operculatus*. The WL-M treatment significantly decreased the SHI, ARFW, SFW, TLA, LFW, and TB in *S*. *cumini*, as well as the A/B in *C. operculatus*, and increased the ARFW, PRFW, TLA, LFW, and TB in *C. operculatus*. Compared with the WL-P treatment, the PRFW and TLA in both species, the LFW in *S*. *cumini*, and the ARFW and TB in *C. operculatus* increased significantly; the A/B in both species significantly decreased.

**Table 1:** Comparative analysis of biomass allocation between *Syzygium cumini* and *Cleistocalyx operculatus* among the treatments.

Species	Treatment	SHI (cm)	ARFW (g)	PRFW (g)	SFW (g)	TLA (cm^2^)	LFW (g)	TB (g)	A/B
*S*. *cumini*	CK-S	30.70 ± 1.45 a ***		12.17 ± 0.74 a ***	56.26 ± 1.23 a ***	816.63 ± 18.38 a ***	22.72 ± 0.56 a ***	91.15 ± 1.84 a ***	6.58 ± 0.39 b **
	WL-S	15.80 ± 1.02 b **	5.14 ± 0.47 a ***	8.37 ± 0.58 bc ***	43.20 ± 1.57 c ***	524.36 ± 11.22 b ***	17.02 ± 0.17 b ***	74.02 ± 2.06 b **	4.43 ± 0.26 d ***
	CK-P	13.60 ± 0.54 b ***		5.20 ± 0.15 d ***	48.26 ± 0.94 b ***	379.15 ± 14.85 d ns	12.35 ± 0.46 d ***	65.81 ± 1.08 c *	11.68 ± 0.22 a **
	WL-P	6.20 ± 0.46 c ns	3.45 ± 0.59 b ***	4.78 ± 0.42 d ***	34.28 ± 1.13 d ***	245.85 ± 3.59 e *	8.28 ± 0.30 e ***	50.77 ± 1.61 d ***	5.33 ± 0.42 c ***
	CK-M	15.30 ± 0.60 b ***		9.35 ± 0.50 b ***	49.07 ± 0.98 b ***	479.31 ± 13.02 c *	15.26 ± 0.28 c **	73.68 ± 1.62 b **	6.94 ± 0.26 b ***
	WL-M	4.00 ± 0.16 c **	2.41 ± 0.25 b ***	7.52 ± 0.45 c ***	31.07 ± 0.60 d ***	347.45 ± 17.50 d ns	12.38 ± 0.31 d ns	53.36 ± 0.87 d ***	4.41 ± 0.14 d ***
*C. operculatus*	CK-S	10.50 ± 0.63 A		29.65 ± 0.52 C	10.58 ± 0.71 AB	425.55 ± 12.60 A	17.74 ± 0.23 A	60.94 ± 0.88 E	1.06 ± 0.04 A
	WL-S	8.4 ± 1.36 AB	18.80 ± 1.55 B	22.73 ± 0.92 D	7.50 ± 1.09 C	303.82 ± 13.64 D	9.84 ± 0.57 D	64.34 ± 1.56 D	0.55 ± 0.04 D
	CK-P	9.9 ± 0.50 A		34.71 ± 1.26 B	8.65 ± 0.54 BC	355.21 ± 11.77 BC	13.72 ± 0.53 BC	60.71 ± 0.61 E	0.78 ± 0.02 B
	WL-P	5.90 ± 0.51 C	17.80 ± 1.20 B	29.62 ± 0.71 C	6.57 ± 0.20 C	316.73 ± 22.08 CD	12.51 ± 0.40 C	74.49 ± 0.78 B	0.57 ± 0.01 D
	CK-M	10.8 ± 0.58 A		41.58 ± 0.59 A	12.57 ± 1.31 A	428.05 ± 8.81 A	14.27 ± 0.48 B	69.26 ± 1.14 C	0.67 ± 0.03 C
	WL-M	6.25 ± 0.62 BC	34.46 ± 1.02 A	32.85 ± 0.59 B	7.88 ± 0.26 C	364.29 ± 6.37 B	12.46 ± 0.62 C	95.89 ± 1.60 A	0.43 ± 0.01 E

Values are means ± SE (n = 5). Different letters above the bars denote significant differences at the P< 0.05 level according to Duncan’s test. Asterisks above the bars denoted statistically significant differences between the species according to the independent-samples t-test (ns, p > 0.05; ∗p< 0.05; ∗∗p< 0.01; ∗∗∗p ≤ 0.001).

SHI, stem height increment; ARFW, adventitious root fresh weight; PRFW, primary root fresh weight; SFW, stem fresh weight; LFW, leaf fresh weight; TLA, total leaf area; TB, total biomass; A/B, the ratio of aboveground fresh weight to belowground fresh weight; CK-S, monocultures under well-watered condition; WL-S, monocultures under waterlogging condition; CK-P, pure planting under well-watered condition; WL-P, pure planting under waterlogging condition; CK-M, mixed planting under well-watered condition; WL-M, mixed planting under waterlogging condition.

### Comparative analyses on the emergence time of AR, AR activity, AR lignin, primary root activity, and primary root porosity between *Syzygium cumini* and *Cleistocalyx operculatus* among the treatments

**Figure 1:**
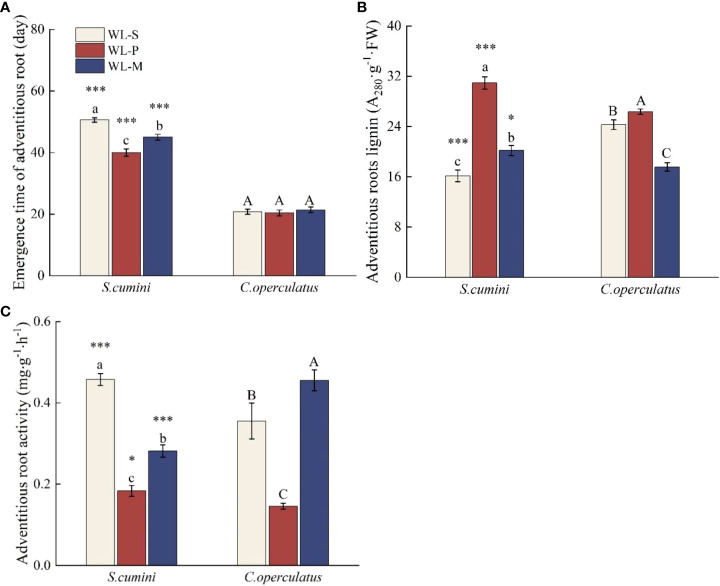
Emergence time of adventitious root **(A)**, adventitious root activity **(B)**, and adventitious root lignin **(C)**, between *Syzygium cumini* and *Cleistocalyx operculatus* among the treatments. CK-S, monocultures under well-watered condition; WL-S, monocultures under waterlogging condition; CK-P, pure planting under well-watered condition; WL-P, pure planting under waterlogging condition; CK-M, mixed planting under well-watered condition; WL-M, mixed planting under waterlogging condition. Values are expressed as means ± SE (*n* = 5). Bars with a different letter within the same species group indicate a significant difference among the treatments at *P*< 0.05, according to ANOVA, followed by Duncan’s test. Asterisks above the bars denote statistically significant differences between the species at *P*< 0.05 according to independent-samples *t*-test (ns, *P* > 0.05; ∗*P*< 0.05; ∗∗∗*P* ≤ 0.001).

As shown in **Figure 1**, the emergence time of ARs under waterlogging of *C*. *operculatus* saplings was significantly shorter than that of *S*. *cumini*, and insignificant differences in the emergence time of ARs were detected among the WL-S, WL-P, and WL-M treatments in *C*. *operculatus*. Compared with the WL-S treatment, the WL-P treatment shortened the emergence time of ARs in *S*. *cumini*, significantly increased the lignin content of ARs, and significantly decreased the AR activity in both species. In addition, compared with the WL-P treatment, the WL-M treatment significantly increased the emergence time of ARs in *S*. *cumini*, as well as the AR activity, but significantly decreased the lignin content in both species.

### Comparative analyses of primary root activity and primary root porosity between *Syzygium cumini* and *Cleistocalyx operculatus* among the treatments

**Figure 2:**
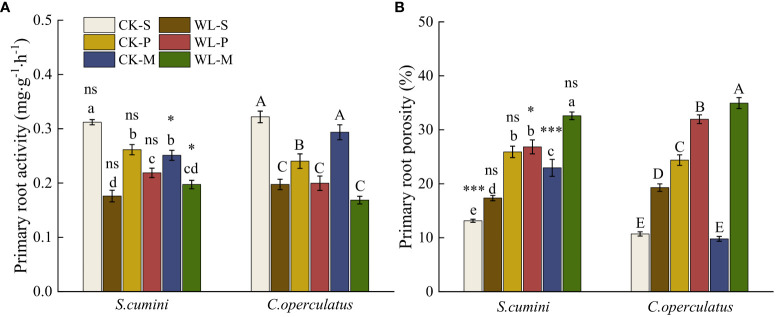
Primary root activity **(A)** and primary root porosity **(B)** between *Syzygium cumini* and *Cleistocalyx operculatus* among the treatments. For abbreviations explanation of treatments are the same as shown in **Figure 1**. Values are expressed as means ± SE (n = 5). Bars with a different letter within the same species group indicate a significant difference among the treatments at *P*< 0.05, according to ANOVA, followed by Duncan’s test. Asterisks above the bars denote statistically significant differences between the species at *P*< 0.05 according to independent-samples t-test (ns, *P* > 0.05; *P < 0.05; ***P ≤ 0.001).

For the primary roots (**Figure 2**), compared with the CK-S treatment, the CK-P treatment significantly decreased the primary root activity and significantly increased the primary root porosity in both species. Meanwhile, the CK-M treatment significantly increased the primary root activity in *C*. *operculatus* and increased the root porosity in *S*. *cumini*. Compared with the CK-P treatment, the CK-M treatment significantly increased the primary root activity of *C. operculatus* and decreased the primary root porosity. Furthermore, compared with the WL-S treatment, the WL-P and WL-M treatments significantly increased the primary root porosity in both species. Compared with the WL-P treatment, the WL-M treatment significantly increased the primary root porosity in both species.

### Comparative analyses of photosynthetic pigments between *Syzygium cumini* and *Cleistocalyx operculatus* among the treatments

**Figure 3:**
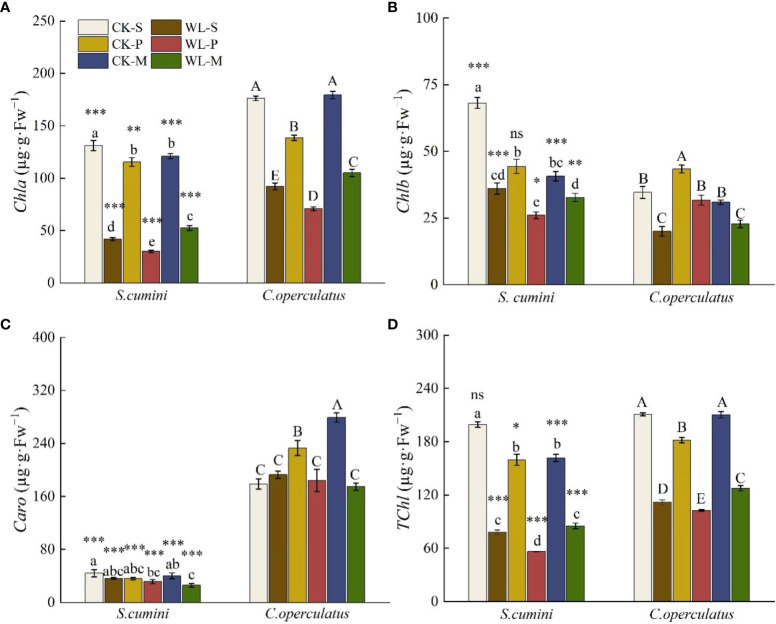
Variations in chlorophyll a (*Chla*) **(A)**, chlorophyll b (*Chlb*) **(B)**, total of chlorophyll (*TChl*) **(C)**, and carotenoids (*Caro*) **(D)** contents between *Syzygium cumini* and *Cleistocalyx operculatus* among the treatments. For abbreviations explanation of treatments are the same as shown in **Figure 1**. Values are expressed as means ± SE (n = 5). Bars with a different letter within the same species group indicate a significant difference among the treatments at *P*< 0.05, according to ANOVA, followed by Duncan’s test. Asterisks above the bars denote statistically significant differences between the species at *P*< 0.05 according to independent-samples t-test (ns, *P* > 0.05; *P < 0.05; **P < 0.05; ***P ≤ 0.001).

Significant differences in the content of chlorophyll a (*Chla*), chlorophyll b (*Chlb*), carotenoids (*Caro*), and the total of chlorophyll (*TChl*) were found in all treatments between *S*. *cumini* and *C*. *operculatus* (except for *TChl* under the CK-M treatment, **Figure 3**). Compared with the CK-S treatment, the CK-P treatment significantly decreased the content of *Chla* and *TChl* in both species and the content of *Chlb* in *S*. *cumini* but significantly increased the content of *Chlb* in *C*. *operculatus*; the CK-M treatment significantly decreased the content of *Chla*, *Chlb*, and *TChl* in *S*. *cumini* but significantly increased *Caro* content in *C*. *operculatus*. Compared with the CK-P treatment, the CK-M treatment significantly increased the content of *Chla*, *Caro*, and *TChl* in *C*. *operculatus*. Furthermore, compared with the WL-S treatment, the content of *Chla* and *TChl* in both species and *Chlb* in *S*. *cumini* significantly decreased under the WL-P treatment, whereas the WL-M treatment significantly increased *Chla* and *TChl* contents. Compared with the WL-P treatment, WL-M significantly increased *Chla* and *TChl* contents in both species, as well as *Chlb* in *S*. *cumini*.

### Comparative analyses on photosynthetic traits and leaf water potential between *Syzygium cumini* and *Cleistocalyx operculatus* among the treatments

**Figure 4:**
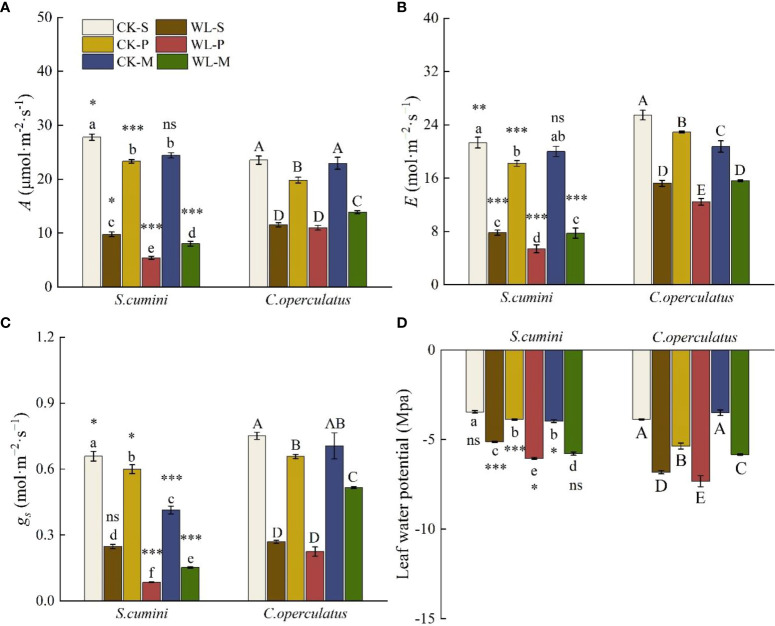
Variations in the leaves’ net photosynthetic rate (*A*) **(A)**, transpiration rate (*E*) **(B)**, stomatal conductance (*g*_s_) **(C)**, and leaf water potential **(D)** between *Syzygium cumini* and *Cleistocalyx operculatus* among the treatments. Values are expressed as means ± SE (n = 5). Bars with a different letter within the same species group indicate a significant difference among the treatments at *P*< 0.05, according to ANOVA, followed by Duncan's test. Asterisks above the bars denote statistically significant differences between the species at *P*< 0.05 according to independent-samples t-test (ns, *P* > 0.05; *P < 0.05; **P < 0.05; ***P ≤ 0.001). For abbreviations explanation of treatments are the same as shown in **Figure 1**.

Significant differences in the net photosynthetic rate (*A*), transpiration rate €, and stomatal conductance (*g*_s_) were found in all treatments (except for *E* under the CK-M treatment and *g*_s_ under the WL-S treatment, **Figure 4**). CK-S treatment showed the highest values of *A*, *E*, and *g*_s_ and the lowest leaf water potential in both species. Compared with CK-S, the CK-P treatment significantly decreased *A*, *E*, *g*_s_, and leaf water potential in both species. Similarly, CK-M significantly decreased *A*, *g*_s_, and leaf water potential in *S*. *cumini*, as well as *E* in *C*. *operculatus*. Compared with the CK-P treatment, CK-M significantly increased *A* and leaf water potential, as well as *A* and *E* in *S*. *cumini*, but significantly decreased *E* in *C*. *operculatus* and *g*_s_ in *S*. *cumini*. Furthermore, compared with the WL-S treatment, the WL-P treatment significantly decreased *A* and *g*_s_ in *S*. *cumini* as well as *E* and leaf water potential in both species. Compared with the WL-P treatment, significant increases in *A*, *E*, *g*_s_, and leaf water potential were observed in both species in the WL-M treatment.

### Comparative analyses on the antioxidant enzymatic activities between *Syzygium cumini* and *Cleistocalyx operculatus* among the treatments

**Figure 5:**
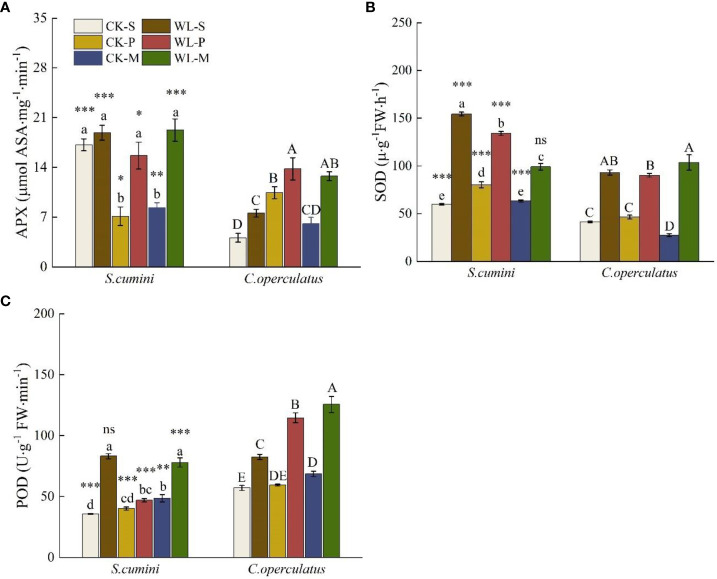
Variations in ascorbate peroxidase (APX) **(A)**, superoxide dismutase (SOD) **(B)**, and peroxidase (POD) **(C)** activities between *Syzygium cumini* and *Cleistocalyx operculatus* among the treatments. Values are expressed as means ± SE (n = 5). Bars with a different letter within the same species group indicate a significant difference among the treatments at *P*< 0.05, according to ANOVA, followed by Duncan’s test. Asterisks above the bars denote statistically significant differences between the species at *P*< 0.05 according to independent-samples t-test (ns, *P* > 0.05; *P < 0.05; **P < 0.05; ***P ≤ 0.001). For abbreviations explanation of treatments are the same as shown in **Figure 1**.

As shown in **Figure 5**, significant interspecific differences in the activities of ascorbate peroxidase (APX), POD, and SOD were found between the two species in all treatments (except for WL-M in SOD and WL-S in POD). Compared with the CK-S treatment, the CK-P treatment significantly decreased the APX activity of *S*. *cumini* but significantly increased that of *C. operculatus*. In addition, the SOD activity of *S*. *cumini* was significantly increased; the CK-M treatment significantly decreased the APX activity of *S*. *cumini* and the SOD activity of *C. operculatus*; the POD activity in both species was significantly increased. Compared with the CK-P treatment, the CK-M treatment significantly decreased the SOD activity in both species and the POD activity of *S*. *cumini*. Furthermore, compared with the WL-S treatment, WL-P and WL-M significantly decreased the SOD and POD activities in WL-P of *S*. *cumini*; however, WL-P and WL-M significantly increased the APX and POD activities of *C. operculatus*. Moreover, compared with the WL-P treatment, WL-M significantly decreased the SOD activity of *S*. *cumini* and increased the SOD activity of *C. operculatus*, as well as the POD activity in both species.

### Comparative analyses on MDA, O_2_^·−^, soluble protein, and proline contents between *Syzygium cumini* and *Cleistocalyx operculatus* among the treatments

**Figure 6:**
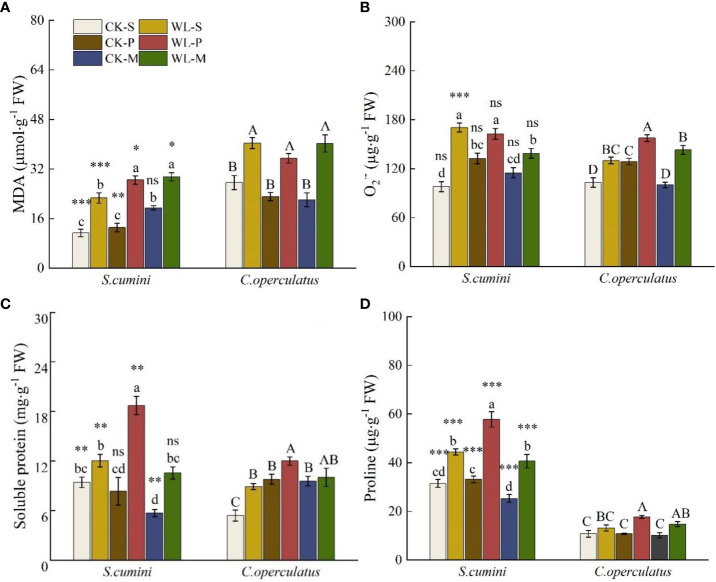
Variations in malondialdehyde (MDA) **(A)**, superoxide radical (O_2_^·−^) **(B)**, soluble protein **(C)**, and proline **(D)** contents between *Syzygium cumini* and *Cleistocalyx operculatus* among the treatments. Values are expressed as means ± SE (n = 5). Bars with a different letter within the same species group indicate a significant difference among the treatments at *P*< 0.05, according to ANOVA, followed by Duncan’s test. Asterisks above the bars denote statistically significant differences between the species at *P*< 0.05 according to independent-samples t-test (ns, *P* > 0.05; *P < 0.05; **P < 0.05; ***P ≤ 0.001). For abbreviations explanation of treatments are the same as shown in **Figure 1**.

As shown in **Figure 6**, compared with the CK-S treatment, the CK-P treatment significantly increased O_2_ **^·−^** of the two species and soluble protein in *C. operculatus*; the CK-M treatment significantly increased the content of MDA in *S*. *cumini* and soluble protein in *C*. *operculatus* but significantly decreased the soluble protein content in *S*. *cumini*. Moreover, compared with the CK-P treatment, the CK-M treatment significantly increased the MDA content in *S*. *cumini* and significantly decreased the O_2_ **^·−^** content in *C*. *operculatus* and the proline content in *S*. *cumini*. In addition, compared with the WL-S treatment, the WL-P treatment significantly increased the MDA content in *S*. *cumini*, the O_2_ **^·−^** content in *C*. *operculatus*, and soluble protein and proline contents in both species; WL-M significantly increased the MDA content in *S*. *cumini* but significantly decreased the O_2_ **^·−^** content in *S*. *cumini*. Compared with the WL-P treatment, WL-M significantly decreased the O_2_ **^·−^** content of the two species, as well as the soluble protein and proline contents in *S*. *cumini*.

### Comparative analysis on non-structural carbohydrate content in the primary roots between *Syzygium cumini* and *Cleistocalyx operculatus* among the treatments

Significant interspecific differences in the levels of soluble sugar, starch, and non-structural carbohydrate contents were found in all treatments (except for soluble sugar in the CK-P treatment, starch in the WL-S and WL-P treatments, and non-structural carbohydrate in the CK-S treatment; **Table 2**). Compared with the CK-S treatment, the CK-P treatment significantly increased the soluble sugar and non-structural carbohydrate contents in *C. operculatus*; CK-M significantly increased the soluble sugar content in *S*. *cumini*, as well as starch and non-structural carbohydrate contents in *C. operculatus*. Compared with the CK-P treatment, soluble sugar and non-structural carbohydrate contents significantly increased in *S*. *cumini*, and the CK-M treatment significantly increased the starch and non-structural carbohydrate contents in *C. operculatus*. In addition, WL-P significantly increased the soluble sugar and non-structural carbohydrate contents in *S*. *cumini*, and the WL-S treatment significantly increased the starch content; the WL-M treatment significantly decreased the soluble sugar, starch, and non-structural carbohydrate contents in *S*. *cumini*, but significantly increased the starch and non-structural carbohydrate contents in *C. operculatus*. Compared with the WL-P treatment, the WL-M treatment significantly decreased the soluble sugar, starch, and non-structural carbohydrate contents in *S*. *cumini* but significantly increased the starch and non-structural carbohydrate contents in *C. operculatus*.

**Table 2:** Variations in soluble sugar, starch, and non-structural carbohydrate contents in the primary root between *Syzygium cumini* and *Cleistocalyx operculatus* among the treatments.

Species	Treatment	Soluble sugar (%)	Starch (%)	Non-structural carbohydrate (%)
*S*. *cumini*	CK-S	12.32 ± 0.83 c *	14.95 ± 1.46 c *	27.27 ± 1.65 cd ns
	WL-S	15.10 ± 1.10 b *	29.52 ± 0.93 a ns	44.62 ± 0.80 b **
	CK-P	12.18 ± 1.02 c ns	12.14 ± 1.47 c ***	24.32 ± 1.21 d ***
	WL-P	25.14 ± 0.97 a ***	24.96 ± 0.99 b ns	50.10 ± 1.71 a ***
	CK-M	15.41 ± 0.69 b **	14.98 ± 0.74 c ***	30.39 ± 1.40 c ***
	WL-M	10.79 ± 0.64 c ns	15.27 ± 0.33 c ***	26.05 ± 0.41 d ***
*C. operculatus*	CK-S	8.95 ± 1.10 B	19.67 ± 0.97 C	28.62 ± 1.19 D
	WL-S	9.54 ± 1.27 AB	26.92 ± 0.93 B	36.44 ± 1.75 BC
	CK-P	12.66 ± 0.77 A	20.79 ± 0.87 C	33.45 ± 0.80 C
	WL-P	10.10 ± 0.51 AB	25.78 ± 1.39 B	35.88 ± 1.69 BC
	CK-M	9.95 ± 1.26 AB	30.04 ± 2.55 B	39.99 ± 2.01 AB
	WL-M	8.65 ± 0.99 B	35.18 ± 1.67 A	43.83 ± 1.80 A

The abbreviations and explanations of treatments, data description, and statistics are the same as those shown in **Table 1**.

### Comparative analyses on total N and total P in the primary roots and ARs between *Syzygium cumini* and *Cleistocalyx operculatus* among the treatments

The ARs of *S*. *cumini* have more total N and P contents than the primary roots (**Table 3**). Compared with the CK-S treatment, total N under the CK-P treatment and total P under the CK-M treatment significantly decreased in *S*. *cumini*. *Cleistocalyx operculatus* significantly increased the total N content in the CK-P and CK-M treatments; compared with the CK-P treatment, CK-M significantly increased the total N content and significantly decreased the total P content in *S*. *cumini*. In addition, compared with the WL-S treatment, the WL-P treatment significantly increased the total N content of the primary roots of *S*. *cumini*, as well as the total P under the WL-M treatment. Meanwhile, WL-M significantly reduced the total N content of the primary roots of *C*. *operculatus*. Compared with the WL-P treatment, the WL-M treatment significantly increased the total N content in *S*. *cumini* but significantly decreased the total N content of the primary roots in *C*. *operculatus* and the total P content in *S*. *cumini*. As for ARs, the WL-M treatment had the lowest total N and total P contents in *S*. *cumini*, whereas the WL-S treatment had the lowest total N and total P contents in *C*. *operculatus*.

**Table 3:** Variations in total N and total P contents in the primary and adventitious root between *Syzygium cumini* and *Cleistocalyx operculatus* among the treatments.

Species	Treatment	Primary roots		Adventitious roots	
		Total N (mg g^−1^)	Total P (mg g^−1^)	Total N (mg g^−1^)	Total P (mg g^−1^)
*S*. *cumini*	CK-S	1.56 ± 0.05 a ***	0.46 ± 0.03 ab **		
	WL-S	0.96 ± 0.05 c ***	0.25 ± 0.02 c ***	2.32 ± 0.07 a **	0.65 ± 0.00 a ***
	CK-P	1.38 ± 0.07 b ***	0.50 ± 0.04 a **		
	WL-P	0.98 ± 0.03 c ***	0.41 ± 0.03 b **	1.96 ± 0.03 b ns	0.59 ± 0.01 b *
	CK-M	1.55 ± 0.15 a *	0.39 ± 0.02 b ***		
	WL-M	1.32 ± 0.10 a ns	0.24 ± 0.01 c ***	2.36 ± 0.05 a ns	0.68 ± 0.02 a ns
*C. operculatus*	CK-S	1.70 ± 0.09 B	0.69 ± 0.06 AB		
	WL-S	1.51 ± 0.07 C	0.55 ± 0.04 B	1.42 ± 0.15 B	0.47 ± 0.02 B
	CK-P	1.99 ± 0.04 A	0.77 ± 0.06 A		
	WL-P	1.55 ± 0.04 BC	0.65 ± 0.06 AB	2.16 ± 0.09 A	0.67 ± 0.01 A
	CK-M	2.02 ± 0.08 A	0.63 ± 0.04 AB		
	WL-M	1.34 ± 0.05 D	0.64 ± 0.03 AB	2.08 ± 0.05 A	0.65 ± 0.02 A

The abbreviations and explanations of treatments, data description, and statistics are the same as those shown in **Table 1**.

### Comparative analyses on the relative competitive intensity, aggressivity, and RII between *Syzygium cumini* and *Cleistocalyx operculatus* among the treatments

**Figure 7:**
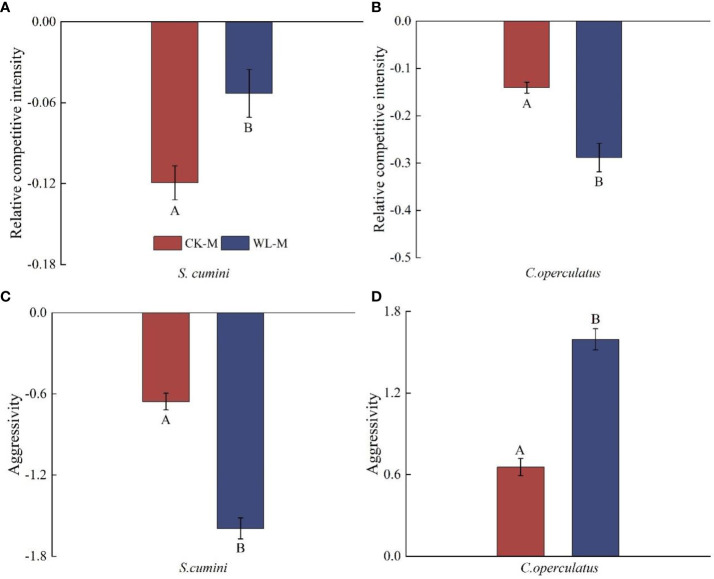
Relative competitive intensity **(A, B)** and aggressivity **(C, D)** between *Syzygium cumini* and *Cleistocalyx operculatus* among the treatments. The abbreviations and explanations of treatments, data description, and statistics are the same as those shown in **Figure 1**.

**Figure 8:**
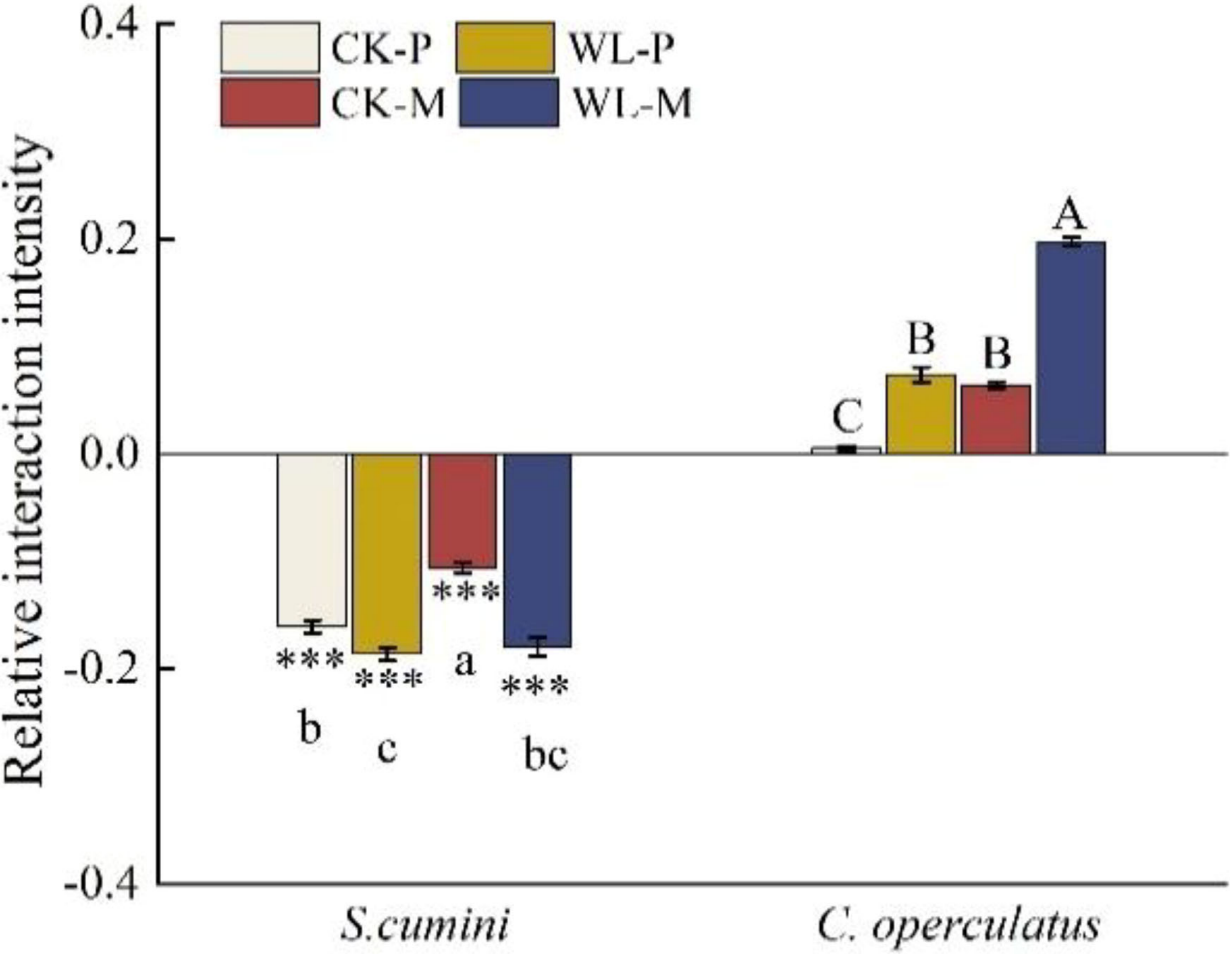
Relative interaction intensity between *Syzygium cumini* and *C. operculatus* among the treatments. For abbreviations explanation of treatments are the same as shown in **Figure 1**. Values are expressed as means ± SE (n = 5). Bars with a different letter within the same species group indicate a significant difference among the treatments at *P*< 0.05, according to ANOVA, followed by Duncan’s test. Asterisks above the bars denote statistically significant differences between the species at *P*< 0.05 according to independentsamples t-test (***P ≤ 0.001).

Under the CK-M and WL-M treatments, the RCI of both species was less than zero; the aggressivity in *S*. *cumini* was less than zero, whereas that in *C. operculatus* was higher than zero (**Figure 7**). Compared with the CK-M treatment, WL-M significantly increased the RCI in *S*. *cumini* and aggressivity in *C. operculatus* but significantly decreased the RCI in *C. operculatus* and aggressivity in *S*. *cumini*. Moreover, as shown in **Figure 8**, significant differences in interaction strength were observed between *S*. *cumini* and *C*. *operculatus* in all treatments; *C*. *operculatus* was always higher than zero, and *S*. *cumini* was consistently less than zero in all treatments. Compared with the CK-P treatment, CK-M significantly increased the interaction strength in both species. In addition, compared with the WL-P treatment, the WL-M treatment significantly increased the interaction strength of *C. operculatus*, and an increasing trend was observed in *S*. *cumini.*

### The SEM of ARs and a comparative comprehensive evaluation between *Syzygium cumini* and *Cleistocalyx operculatus* among different treatments following the planting pattern

**Figure 9:**
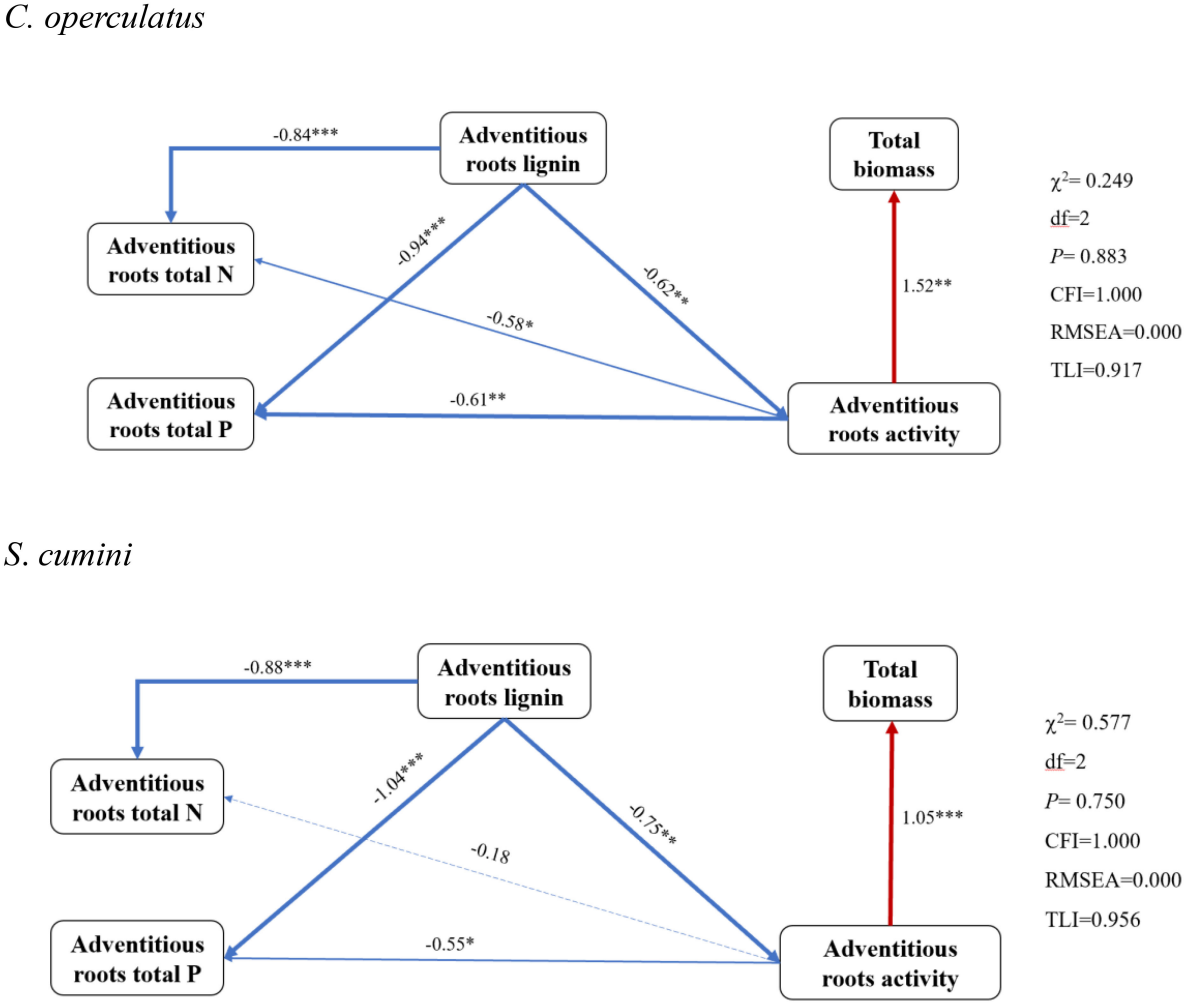
The SEM of adventitious roots of the two species. The solid blue line indicates a negative correlation, the solid red line indicates a positive correlation, and the dashed line indicates no significant correlation. The thickness of the line indicates the degree of correlation.

The SEM shows the relationship among ARs’ nitrogen, phosphorus, and lignin contents and root activity (**Figure 9**). We used TB as the plant’s response to competition and stress. As shown by the SEM, ARs’ lignin content was significantly and negatively correlated with nitrogen and phosphorus contents in both species. Similarly, ARs’ activity was also negatively correlated with nitrogen and phosphorus contents (more significantly in *C*. *operculatus*) but significantly and positively correlated with TB. Morphological and physiological traits were reduced to four principal components that explained 87.44% of the variance (**Table 4**). *Syzygium cumini* had higher CE values than *C*. *operculatus* in the CK-S and WL-S treatments. Compared with the CK-S, CK-P, and CK-M treatments, the CE values of *S*. *cumini* under the WL-S, WL-P, and WL-M treatments showed a greater tendency to decrease than *C*. *operculatus*. Moreover, *C*. *operculatus* always had higher CE values than *S*. *cumini* under the WL-P and WL-M conditions.

**Table 4:** Comparative analyses of the value of the principal component [*C* (m)], membership function value [*M* (m)], and comprehensive evaluation value (*CE*) of *S*. *cumini* and *C. operculatus* among all treatments.

Species	Treatment	*C* (1)	*C* (2)	*C* (3)	*C* (4)	*M* (1)	*M* (2)	*M* (3)	*M* (4)	*CE*
*S*. *cumini*	CK-S	1.13898	1.32065	1.24222	0.61869	0.950	1.000	0.932	0.758	0.944
	WL-S	−1.06413	0.84815	1.03652	0.94665	0.217	0.845	0.860	0.853	0.513
	CK-P	0.68539	0.82637	0.33253	−1.04451	0.799	0.838	0.614	0.276	0.740
	WL-P	−1.71556	1.08938	−1.42271	1.19726	0.000	0.924	0.000	0.926	0.315
	CK-M	0.52934	0.70447	0.73284	−0.60572	0.748	0.798	0.754	0.403	0.731
	WL-M	−0.93426	0.46417	−0.64589	−1.99545	0.260	0.719	0.272	0.000	0.355
*C. operculatus*	CK-S	1.28764	−0.21754	−1.36725	0.00909	1.000	0.495	0.019	0.581	0.712
	WL-S	−0.37024	−0.91099	−0.3913	−0.24421	0.448	0.268	0.361	0.508	0.397
	CK-P	0.72761	−0.59416	−0.66743	−0.13773	0.814	0.372	0.264	0.539	0.609
	WL-P	−0.63376	−1.13981	0.45854	−0.66255	0.360	0.193	0.658	0.386	0.358
	CK-M	0.95469	−0.66241	−0.744	1.45421	0.889	0.350	0.237	1.000	0.680
	WL-M	−0.60568	−1.72828	1.43593	0.46426	0.370	0.000	1.000	0.713	0.386

The abbreviations and explanations of treatments are the same as those shown in **Table 1**.

## Discussion

Although ecologists have primarily emphasized competition as a pivotal process in forest communities, the significance of facilitation cannot be undermined, particularly in certain ecological niches exposed to drastic environmental changes. The magnitude and nature of interactions among different species may be influenced by environmental factors and species tolerance to abiotic stresses. This knowledge is essential for ensuring the long-term success and sustainability of planted forests in a changing climate, thereby promoting forest conservation and ecosystem restoration.

### Under well-watered conditions, the intraspecific interaction of *Syzygium cumini* in the pure planting pattern was negative compared with that of *Cleistocalyx operculatus*

Different plants have different ways of coping with intraspecific competition. We found that well-watered pure planting treatment affects the growth and physiology of both species. Closely related individuals growing in close proximity may engage in more intense competition because of their similar genotypes and patterns of resource utilization (Young, 1981). In addition, for the root system, according to Mahall and Callaway (1991), individuals of this species strongly reduced their root growth (**Table 1**) when these roots encountered the roots of another species individual (Mahall and Callaway, 1992). However, *C*. *operculatus* increased the fresh weight of the primary roots under well-watered pure planting conditions, but it is not a so-called “tragedy of the commons” in *C*. *operculatus*, which has a better trade-off strategy under intraspecific competition, as we found that in two-thirds of the treatments the size of the roots of two *C*. *operculatus* in the same pot produced a difference in biomass, thereby avoiding competition for limited resources (**Table 1**). Moreover, *C*. *operculatus* exhibited elevated nitrogen levels (**Table 3**). An optimal water–nitrogen (N) supply can enhance plant biomass accumulation through the improvement of root growth (Chen et al., 2018). Therefore, our results demonstrate that *C*. *operculatus* exhibits superior adaptation to intraspecific competition compared with *S*. *cumini*.

### Interspecific interactions are beneficial for both species under well-watered conditions

Montesinos-Navarro et al. (2019) have reported that reducing the competition among distantly related plants is a prevalent mechanism enabling plant coexistence. *Syzygium cumini* possesses a larger leaf area and LFW (**Table 1**), providing a larger photosynthetic area for the accumulation of resources and energy necessary for growth. In addition, *C*. *operculatus* exhibits heightened primary root activity (**Figure 2**) and leaf water potential (**Figure 4**), which aids water absorption from the soil (Samui and Kar, 1981), and improves photosynthesis (**Figure 4**) to promote its growth. In the presence of non-self and non-kin neighbors, certain plant species exhibit an enhanced root allocation at the cost of reproductive investment, suggesting their ability to recognize neighbor identity at the root level (Chen et al., 2012). Dudley and File (2007) suggested that plant roots play a role in the “kin recognition” of neighboring species through the exchange of recognition substances such as root exudates and potentially common mycorrhizal networks (Anten and Chen, 2021). Although *C*. *operculatus* increased the fresh weight of the primary roots, no significant change in N uptake capacity was observed, a phenomenon that has been explained as a foraging strategy of the plant (Zhang et al., 2016). Although *S. cumini* and *C. operculatus* belong to the Myrtaceae family, their genetic relatedness is not as close as that within the same species. Mixed planting separates their respective relatives, thereby reducing resource use similarity. Furthermore, mixed planting with diverse neighbors alleviates the competitive pressure on both species (**Figures 7**, **8**).

### Mixed planting alleviates the negative effects of waterlogging on both species, compared with the pure planting pattern

Waterlogging primarily affects the root system of plants at the onset. ARs are important morphologically adaptable characteristics of plant response to waterlogging, which contribute to O_2_ diffusion, as well as nutrient and water uptake, and reduce the accumulation of toxic substances (Zhang et al., 2015). In this study, we observed the formation of ARs in *S*. *cumini* and *C*. *operculatus* under waterlogged conditions. In addition, *C*. *operculatus* exhibited a greater number of ARs that emerged at a shorter time compared with *S*. *cumini* (**Figure 1**). SEM analysis revealed a more significant correlation between the activity of ARs and their nitrogen and phosphorus contents in *C*. *operculatus* than in *S*. *cumini* (**Figure 9**), indicating that ARs of *C*. *operculatus* could play a greater role. Moreover, the significant increase in APX and POD activities and *Caro* content in *C*. *operculatus* (**Figures 3**, **5**) suggested that this species could enable the efficient removal of a substantial amount of H_2_O_2_ and protect the photosynthetic apparatus against ROS (Liao et al., 2019; Hasanuzzaman et al., 2020). These phenomena may be related to the increase of lignin and the reduction of root activity that were more pronounced in the ARs of *S*. *cumini* compared with *C*. *operculatus* (**Figure 1**), thereby facilitating the increase of oxygen exchange between the root system and water (Ayi et al., 2016). Consequently, waterlogging intensifies intraspecific competition in *S*. *cumini*, while *C*. *operculatus* demonstrates superior adaptation to waterlogging stress.

The growth of both species was clearly promoted under waterlogging conditions in the mixed planting pattern (**Table 4**). It significantly mitigated the negative effects of flooding on photosynthesis in both species. In addition, both species exhibited a notable increase in AR activity (**Figure 1**). This enhanced activity facilitated the transportation of oxygen and nutrients, effectively reducing peroxide accumulation (**Figure 6**). Moreover, starch in the plant roots serves as a reserve material, providing sugars for the anoxic metabolism of the root system when the plant is subjected to waterlogging (Irfan et al., 2010). Prolonged waterlogging inhibits plant respiration caused by anoxia, thereby reducing ATP production. In response to this condition, water-tolerant plant roots gradually decrease their consumption of soluble sugars and convert them into complex sugars, particularly starch, for storage. This adaptation aids in maintaining appropriate energy metabolism in the roots. The increase in carbohydrate content has been attributed to the high waterlogging tolerance (Angelov et al., 1996). *Cleistocalyx operculatus*, being a more WL-tolerant species, exhibits increased root starch content under the mixed planting pattern (**Table 2**). Conversely, pure planting of *S*. *cumini* hinders its photosynthesis and leads to the accumulation of a high carbohydrate content in the roots (**Table 2**), possibly because of a more severe inhibition of aerobic respiration in the primary root system, which hampers carbohydrate consumption (Wu et al., 2022). Moreover, the accumulation of high levels of carbohydrates under stress could be induced by reduced root growth (Araki et al., 2012). The mixed planting pattern promotes the consumption of carbohydrates by *S*. *cumini* to meet its growth requirements. Therefore, the mixed planting pattern under waterlogging conditions is beneficial for the growth of both species.

## Conclusion

In the pure planting pattern, intraspecific competition occurs within *S*. *cumini*, which is further intensified by waterlogging. Conversely, the competition between *C. operculatus* species in the pure planting pattern was less pronounced. The ecological niche differentiation of the two species enables them to utilize different resources and occupy distinct niches because of species affinities, which enhances their growth. The mixed planting system between the two species relieved the pressures of intraspecific competition under waterlogging, thereby improving their tolerance to waterlogging.

## Data availability statement

The raw data supporting the conclusions of this article will be made available by the authors, without undue reservation.

## Author contributions

MT: Data curation, Formal Analysis, Investigation, Methodology, Writing – original draft. DL: Investigation, Writing – review & editing. E-HC: Data curation, Formal Analysis, Writing – review & editing. LM: Writing – review & editing, Investigation, Methodology. WY: Investigation, Writing – review & editing. JZ: Investigation, Writing – review & editing. BC: Investigation, Writing – review & editing. LL: Investigation, Writing – review & editing. HT: Investigation, Writing – review & editing. BY: Investigation, Writing – review & editing. FY: Writing – review & editing, Funding acquisition, Supervision.
